# Cell Line Specific Modulation of Extracellular Aβ_42_ by Hsp40

**DOI:** 10.1371/journal.pone.0037755

**Published:** 2012-05-29

**Authors:** Anna Carnini, Lucas O. M. Scott, Eva Ahrendt, Juliane Proft, Robert J. Winkfein, Sung-Woo Kim, Michael A. Colicos, Janice E. A. Braun

**Affiliations:** 1 Department of Physiology and Pharmacology, Hotchkiss Brain Institute, University of Calgary, Calgary, Alberta, Canada; 2 Department of Biochemistry and Molecular Biology, Southern Alberta Cancer Research Institute, University of Calgary, Calgary, Alberta, Canada; National Institutes of Health, United States of America

## Abstract

Heat shock proteins (Hsps) are a set of molecular chaperones involved in cellular repair. They provide protective mechanisms that allow cells to survive potentially lethal insults, In response to a conditioning stress their expression is increased. Here we examined the connection between Hsps and Aβ_42_, the amyloid peptide involved in the pathological sequence of Alzheimer’s disease (AD)_._ Extracellular Aβ_42_ associates with neuronal cells and is a major constituent of senile plaques, one of the hallmarks of AD. Although Hsps are generally thought to prevent accumulation of misfolded proteins, there is a lack of mechanistic evidence that heat shock chaperones directly modulate Aβ_42_ toxicity. In this study we show that neither extracellular Aβ_42_ nor Aβ_42/_PrP^C^ trigger the heat shock response in neurons. To address the influence of the neuroprotective heat shock response on cellular Aβ_42_, Western analysis of Aβ_42_ was performed following external Aβ_42_ application. Five hours after a conditioning heat shock, Aβ_42_ association with CAD cells was increased compared to control neurons. However, at forty-eight hours following heat shock Aβ_42_ levels were reduced compared to that found for control cells. Moreover, transient transfection of the stress induced Hsp40, decreased CAD levels of Aβ_42_. In contrast to CAD cells, hippocampal neurons transfected with Hsp40 retained Aβ_42_ indicating that Hsp40 modulation of Aβ_42_ proteostasis is cell specific. Mutation of the conserved HPD motif within Hsp40 significantly reduced the Hsp40-mediated Aβ_42_ increase in hippocampal cultures indicating the importance of this motif in regulating cellular Aβ_42_. Our data reveal a biochemical link between Hsp40 expression and Aβ_42_ proteostasis that is cell specific. Therefore, increasing Hsp40 therapeutically with the intention of interfering with the pathogenic cascade leading to neurodegeneration in AD should be pursued with caution.

## Introduction

Alzheimer’s disease (AD), an age-dependent neurodegenerative disease that is estimated to affect 35 million people worldwide is characterized by amyloid deposits, neurofibrillary tangles, selective neuronal loss, cognitive decline and memory loss [1], [2]. Multiple lines of evidence suggest an imbalance between the production and clearance of Aβ_1−42_, a 42 residue long β amyloid protein that spontaneously self aggregates into dimers, oligomers, protofibrils, and fibrils and initiates a toxic sequence of events leading to synaptic dysfunction and dementia [3]. Aβ_42_ as well as Aβ_40_ are derived from the amyloid precursor protein (APP) by the sequential proteolytic processing of α, β and γ secretases (reviewed: [4], [5]. Following proteolysis, the peptides can be secreted or transferred to the endosomal/lysosomal system. Intraneuronal Aβ_42_ is comprised of both uptake of Aβ_42_ from the extracellular space as well as intracellular cleavage of APP [6]. Synaptic activity increases levels of secreted, extracellular Aβ peptides while reducing intracellular levels [7].

Why do the physiological mechanisms that under normal circumstances tightly regulate Aβ_42_ production, cell association and clearance fail? Deficiencies in cellular chaperone systems are one possibility. Molecular chaperones maintain protein homeostasis by assisting nascent polypeptides to fold, protecting mature proteins from stresses and eliminating misfolded proteins. Protein quality control mechanisms are critical to neural function and defects in proteolytic pathways are widely held to lead to neurodegeneration [8]. The cellular level of chaperones might affect the toxicity of Aβ_42._ In fact, enhancement of the cellular quality control machinery, has been proposed to prevent or delay the cascade of misfolding in conformational diseases [9], [10]. In addition to maintenance of protein homeostasis (proteostasis) by constitutive chaperones, in response to a number of stressful stimuli, there is an induction of stress-induced chaperones (eg Hsp40, Hsp90, Hsp70 and Hsp27). Understanding the biochemical sequence of events that underlies Aβ_42_-mediated neurodegeneration requires a clear understanding of the role(s) that chaperones play in the AD pathogenic cascade.

A number of chaperones are implicated in Aβ_42_ proteostasis [11]. Several chaperones have been found both in association with senile plaques [12]–[14] as well as endogenous Aβ_42_ [15]. These reports have given rise to the notion that molecular chaperones are suppressors of toxic Aβ_42_ conformations leading to AD. This idea is consistent with the observations that heat shock genes appear to be induced poorly late in life and that the principal risk factor for AD is age [9]. Further support for this view has come from reports demonstrating that in experimental models, Hsp70 [16], [17], Hsp27 [18] and Hsp90 [16] protect against the toxic effects of Aβ_42_. Also, Hsp70 is reported to suppress cognitive deficits and pathological phenotypes in AD mice [19]. *In vitro* Hsp70/40 and Hsp90 suppressed early stages of Aβ_42_ assembly into aggregates but had no effect on fibrils [16]. Still, many questions remain unanswered regarding the chaperone folding paths for Aβ_42_. For example, Mearow and colleagues have shown that heat shock of neonatal rat cortical cultures increases the detrimental effects of Aβ_42_ on cell survival while overexpressing Hsp27 protects against Aβ_42_ [18]. In mice models of Alzheimer’s disease overall content of the chaperone αβ crystallin is reduced [20]. However, in contrast to the concept of therapeutic rescue by chaperones, several molecular chaperones actually support the formation of the toxic Aβ_42_ oligomeric species [21]–[23]. This promotion of Aβ_42_ oligomerization by select chaperones has similar features to that observed in response to general anesthesia [24], [25].

In addition to cellular chaperones, cellular prion protein (PrP^C^)/Aβ_42_ association could influence Aβ_42_ quality control. Aβ_42_ in contrast to Aβ_40,_ associates rapidly with neuronal cells [26]. Two distinct Aβ_42,_ oligomeric conformations accumulate on the surface of living cells [27]. Exposure of Aβ_42_ to pH = 6 for 24 hours to mimic endosomal conditions increases Aβ_42_ binding to PC12 cells [28]. The cellular prion protein has been shown to act as a functional high affinity receptor for Aβ_42_ [29]–[31] Strittmatter and colleagues report that association of PrP^C^ with Aβ_42_ mediates downstream Aβ_42_-impairement of hippocampal long term potentiation and that AD transgenic mice lacking PrP^C^ accumulate Aβ_42_ but have normal survival and test normal for learning and memory [29], [31]. Furthermore, transgenic overexpression of PrP^C^ is shown to enhance amyloid plaque formation in an AD mouse model [32]. Along these lines, pathological levels of Aβ_42_ have been shown to disrupt PrP^C^ modulation of NMDA (N-Methyl-d-aspartate) receptor activity [33]. However, in contrast, Balducci et al report that Aβ_42_ impairs consolidation of long-term recognition memory in mice independent of PrP^C^, raising questions regarding the role of Aβ_42_/PrP^C^ association in AD progression [30]. Additionally, other molecules (eg STI1) are recognized to bind PrP^C^, but whether these agents compete with Aβ_42_ for binding is unknown [34]. Following cell association, insoluble Aβ_42_ aggregates localize to endosome/lysosome compartments [35], [36]. Curiously, one report reveals that PrP^C^ inhibits β-secretase cleavage of amyloid precursor protein and reduces Aβ_42_ levels [37].

That said, which molecular chaperones directly regulate Aβ_42_ protestasis and/or toxicity remain to be established. In this study we have monitored the association of Aβ_42_ with cultured neural cells following induction of the heat shock. Our findings demonstrate that heat shock initially increases Aβ_42_ association with CAD neuroblastoma cells but is followed by a decline in cellular Aβ_42_ levels at 48 hours. Transient transfection experiments revealed that Hsp40 decreased cellular levels of Aβ_42_ in CAD cell but increased cellular levels of Aβ_42_ in hippocampal cultures. We evaluated the influence of exogenously applied soluble PrP^C^ on cellular uptake/processing of Aβ_42_ to test the hypothesis that soluble PrP^C^ would bind to Aβ_42_ and reduce association with cell anchored PrP^C^, thereby blocking an early event in the Aβ_42_ pathogenic cascade. Here we document that PrP^C^ failed to decrease cellular association of Aβ_42_. Our data reveal a biochemical link between cellular levels of Hsp40 and Aβ_42_ that is cell line specific. This raises the possibility that Hsp40 is involved in the pathogenic cascade leading to dementia and neurodegeneration in AD.

## Results and Discussion

### Extracellular Aβ_42_ does not Trigger the Heat Shock Response

**Figure 1:**
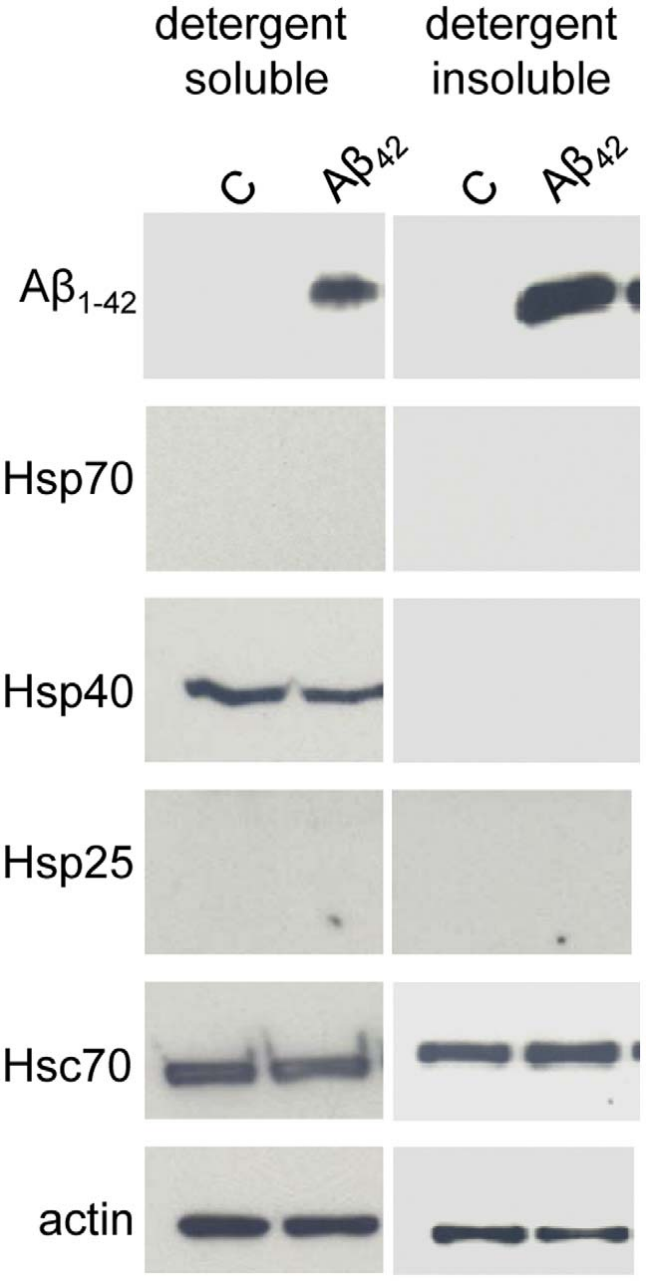
Aβ_42_ does not trigger the heat shock response in CAD neural cells. CAD cells were incubated with 25 µM Aβ_42_ for 48 hours, washed in PBS and lysed. 30 µg of soluble protein or 10 µl of the insoluble fraction were resolved by SDS-PAGE and the indicated proteins were evaluated by Western blot analysis. β-actin is shown as a loading control. Data are representative of 3 separate experiments.

Heat shock chaperones are induced in response to a number of cell stressors such temperature, ischemia and heavy metals. During aging when heat shock genes are thought to be induced poorly, humans are susceptible to AD. To gain insight into the involvement of the heat shock response in Aβ_42_ pathogenic cascades, we carried out biochemical studies to establish whether the treatment of neuroblastoma cells with Aβ_42_ triggers the expression of the stress-induced chaperones. Mouse CAD neuroblastoma cells were incubated with a high concentration (25 µM) of Aβ_42_ for 48 hours, rinsed in PBS and solublized. 30 µg of supernatant (1% TX-100/0.1% SDS soluble proteins) and 10 µl of total pellet (1% TX-100/0.1% SDS insoluble proteins) were subjected to Western analysis. **Figure 1** demonstrates that Aβ_42_ was clearly found in both soluble and insoluble CAD cell fractions. The expression of cellular Hsp70 (heat shock protein of 70 kDa), Hsp25 (Heat shock protein of 25 kDa) and Hsp40 (Heat shock protein of 40 kDa) in CAD cells did not change following treatment with Aβ_42_ for 48 hours. Hsp70 and Hsp25 were not detectable in either the presence or absence of Aβ_42_. Moreover, Hsp40 showed modest expression in control CAD cells as previously described [38], and no change was observed in response to Aβ_42_ treatment. The expression levels of the constitutive chaperone Hsc70 (Heat shock cognate protein of 70 kDa) also did not change in response to Aβ_42_. Actin is shown as a loading control.

### Aβ_42_ does not Block the Heat Shock Response

**Figure 2:**
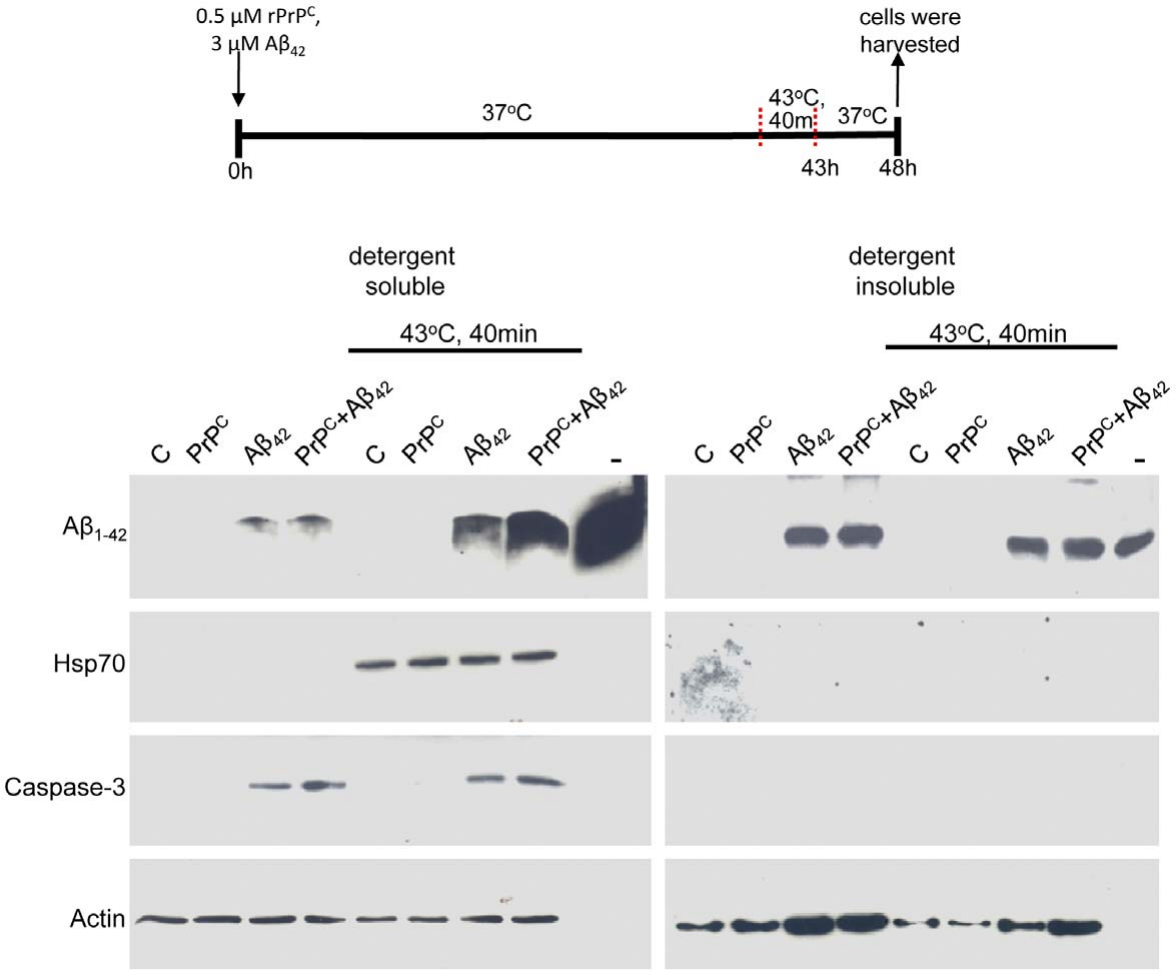
Heat shock initially increases Aβ_42_ association with CAD neural cells. In four independent experiments CAD cells were incubated with 3 µM Aβ_42_ and/or 0.5 µM bovine rPrP^C^, after ∼42 hours cells were subjected to heat-shock at 43°C for 40 minutes and then allowed to recover for 5 hours. The cells were rinsed in PBS and lysed. 30 µg of the soluble protein or 10 µl of the insoluble fraction was resolved by SDS-PAGE and subjected to Western blot analysis. Data are representative of 4 separate experiments.

The heat shock response is a highly conserved cell survival program that enhances cell survival to subsequent insults [9]. Since interference with the heat shock response would be expected to reduce protein surveillance and triage mechanisms and downstream cell survival, we next tested whether Aβ_42_ altered induction of the heat shock chaperones. CAD cells were incubated with 3 µM Aβ_42_ then heat shocked at 43°C for 40 min and allowed to recover for 5 hours prior to lysis and Western analysis. **Figure 2** clearly shows that Hsp70 is induced by heat shock and that Aβ_42_ does not alter the induction of this stress inducible chaperone. As expected, Aβ_42_ treatment of CAD cells triggers apoptotic pathways as shown by activation of caspase 3, a marker of programmed cell death. The Aβ_42_ activation of caspase 3 was not altered by heat shock. Heat shock treatment increased cellular levels of soluble Aβ_42_ ∼3.5 fold, suggesting that following heat shock neurons are more prone to the cellular toxicity of Aβ_42_ **(** **Figure 2** **)**. This observation is in line with a previous study demonstrating that Aβ_42_ decreases cortical neuron cell survival and that heat shock renders neurons more vulnerable to Aβ_42_ treatment [18]. Taken together, our data demonstrate that, although a large number of stressors activate the neuroprotective heat shock response, Aβ_42_ failed to increase the expression of the heat shock chaperones. Moreover, removal/disruption of the protective effects of a conditioning heat shock against cell death is not part of the Aβ_42_ pathogenic cascade.

### Soluble PrP^C^ does not Block Cell Association of Aβ_42_

**Figure 3:**
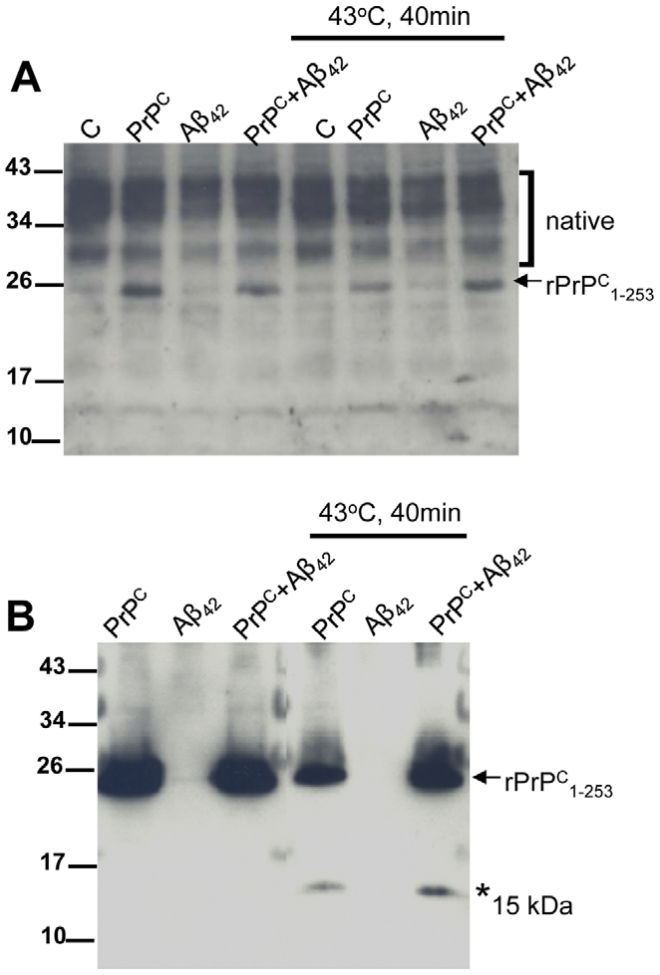
PrP^C^ associates with CAD cells. (**A**) CAD cells were incubated with 3 µM Aβ_42_ and/or 0.5 µM bovine rPrP^C^, after ∼42 hours cells were subjected to heat-shock at 43°C for 40 minutes and then allowed to recover for 5 hours. The cells were rinsed in PBS and lysed. 30 µg of the soluble protein or 10 µl of the insoluble fraction was resolved by SDS-PAGE and cellular PrP^C^ was determined by Western analysis. (**B**) Purified rPrP^C^ was added to DMEM/F12 tissue culture media, subjected to heat-shock (43°C for 40 minutes) and then incubated at 37°C for 48 hours in the absence of cell lines and dissolved directly in sample buffer.

PrP^C^ has been proposed to act as a functional high affinity receptor for Aβ_42_ [29]–[31]. Based on these reports, we wanted to determine how PrP^C^ may impact the cellular association of Aβ_42_. Therefore, we examined the possibility that soluble recombinant PrP^C^ would competitively displace Aβ_42_ from CAD cells thereby reducing the association of Aβ_42_ with the neuronal cell culture. Treatment with 0.5 µM recombinant PrP^C^ did not induce Hsp70 or activation of caspase 3. Also, PrP^C^ failed to alter the heat shock induction of Hsp70 or the Aβ_42_ induced activation of caspase 3. No difference in cellular Aβ_42_ association was observed between Aβ_42_ and PrP^C^/Aβ_42_ treated control (no-heat shock) cells. In fact, rather than inhibit cellular association of Aβ_42,_ PrP^C^/Aβ_42_ co-treatment followed by heat shock revealed that PrP^C^ increased Aβ_42_ levels in the soluble CAD cell fractions (**Figure 2**). CAD cells express endogenous PrP^C^, a glycosylphosphatidyl (GPI) anchored plasma membrane protein [39], [40] (**Figure 3**) that is subject to N-linked glycosylation and non-, mono- and di-glycosylated versions of PrP^C^ simultaneously exist [41]. Recombinant bovine PrP^C25−232^ migrated further on SDS-PAGE then native unglycosylated PrP^C^ therefore rendering cell association of exogenous recombinant PrP^C^ distinguishable from endogenous PrP^C^. Exogenous recombinant PrP^C^ was observed to associate with cells (**Figure 3**). In the absence of CAD cells recombinant PrP^C^ was observed to undergo partial breakdown following heat shock for 40 min at 43°C. Taken together our results show that exogenous PrP^C^ does not block cellular Aβ_42_ association, in fact, following heat shock PrP^C^/Aβ_42_ co-treatment increased cell associated Aβ_42_.

### Heat Shock Promotes Time Dependent Clearance of Aβ_42_

**Figure 4:**
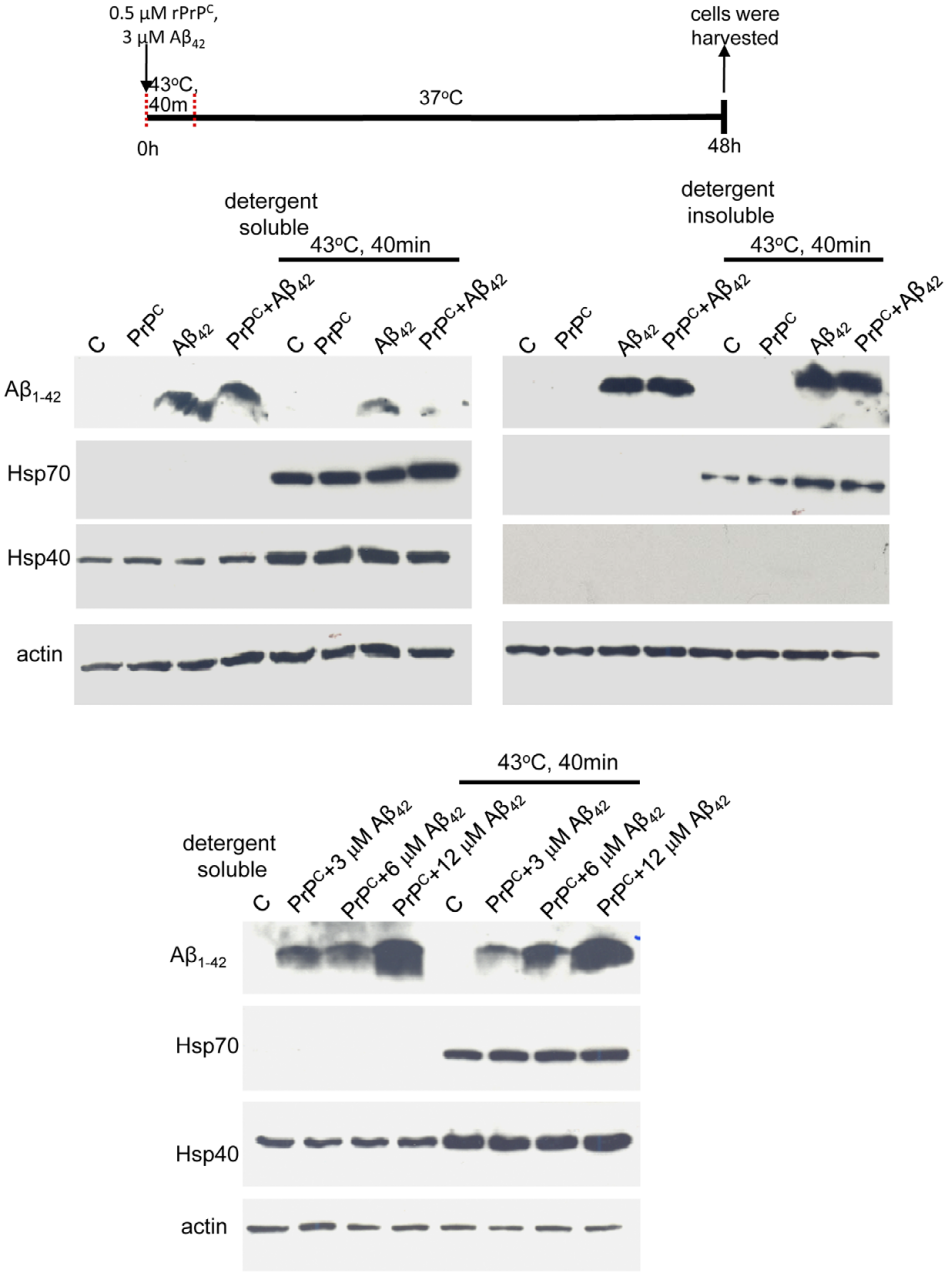
Aβ_42_ levels are reduced 48 hours following induction of the cellular heat shock response. CAD cells were incubated with 3 µM Aβ_42_ and/or 0.5 µM bovine rPrP^C^. Immediately following addition of Aβ_42_ or rPrP^C^, the cells were subjected to heat-shock at 43°C for 40 minutes, allowed to recover for 48 hours, and washed in PBS prior to lysis. 30 µg of soluble cellular protein or 10 µl of the insoluble fraction was resolved by SDS-PAGE and subjected to Western blot analysis. Lower panel: CAD cells were incubated with the indicated increasing concentrations of Aβ_42_ and/or 0.5 µM mouse (L42) rPrP^C^. Data are representative of 3 separate experiments.

Although heat shock facilitated the pathogenicity of Aβ_42_ as measured by its increased cellular association, we speculated that this may be due to the physical effects of heat shock on membrane permeability rather than the conformational processing of PrP^C^ by stress induced chaperones. To gain further insight into the relationship between cellular uptake/clearance of Aβ_42_ and the heat shock response, we carried out immunoblot analysis on CAD cells in which the heat shock was given at an earlier time point thereby increasing the time Aβ_42_ is exposed to the stress-induced chaperones. **Figure 4** shows that when a 40 min heat shock was given starting at the time that Aβ_42_ was applied to CAD cells, the cellular levels of soluble Aβ_42_ at the 42 hour time point was reduced. Aβ_42_ does not always resolve as a discrete band by SDS-PAGE depending on abundance and the characteristic wide Aβ_42_ band is shown in Figure 4. Cellular levels of Hsp70 and Hsp40, which are elevated 3 hours following heat shock [38], remained elevated 48 hours following heat shock and at the 48 time point translocation of Hsp70 (but not Hsp40) to the detergent insoluble fraction was observed. Our observations demonstrate that while soluble Aβ_42_ is increased 5 hours following heat shock (**Figure 2**), soluble Aβ_42_ is decreased 48 hours following heat shock (**Figure 4**) indicating that with time heat shock chaperones increase cellular Aβ_42_ clearance. Geldanamycin-treatment of CAD cells which induces Hsp40 but not Hsp70 [38] was also observed to reduce cellular levels of Aβ_42_ (data not shown)_._ **Figure 4** **(lower panel)** clearly shows that CAD cell levels of Aβ_42_ increase in response to increasing Aβ_42_ concentrations. Again, PrP^C^, (either bovine upper panel or mouse lower panel **Figure 4**) did not reduce cell association of Aβ_42_ and did not alter the heat shock response.

**Figure 5:**
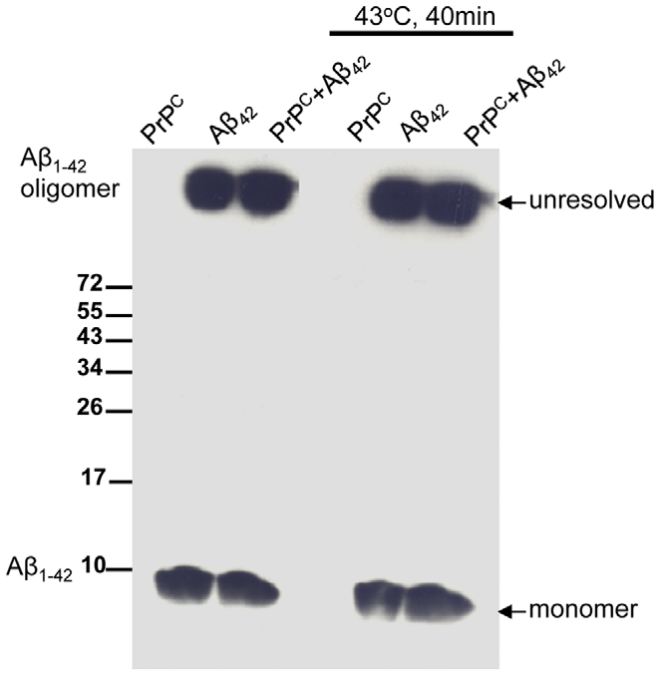
Aβ_42_ is not directly altered by heat shock. (**A**) Purified Aβ_42_ was added to DMEM/F12 tissue culture media, subjected to heat-shock (43°C for 40 minutes) and then incubated at 37°C for 48 hours in the absence of cell lines and dissolved directly in sample buffer.

**Figure 6:**
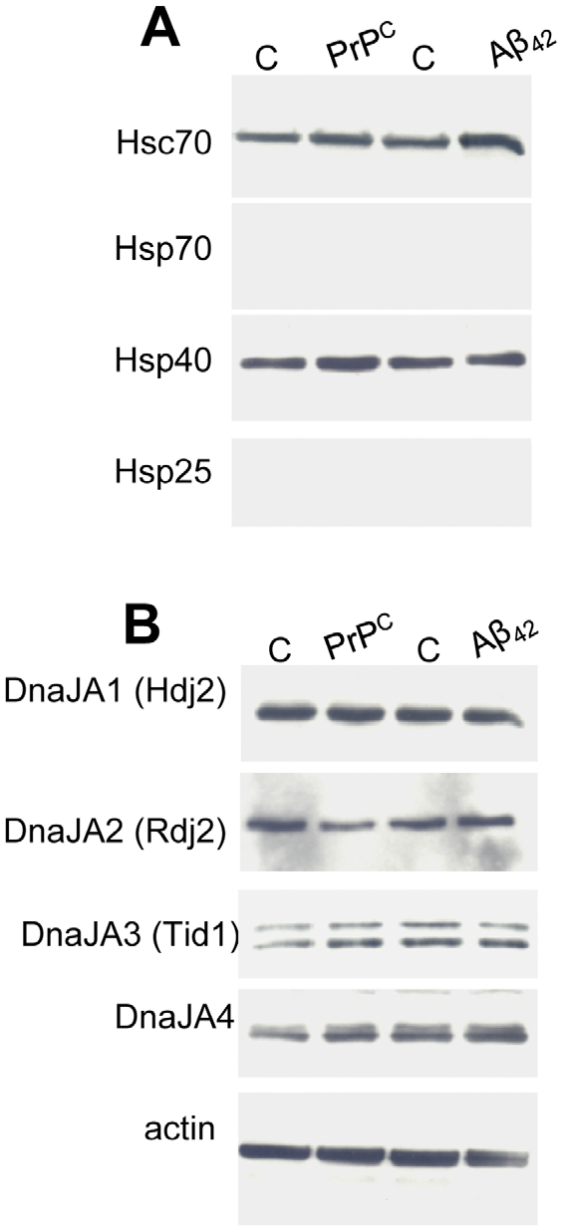
Aβ_42_ or bovine rPrP^C^ do not alter expression of the J proteins DnaJA1(Hdj2), DnaJA2(Rdj2), DnaJA3 (Tid1) or DnaJA4 in neural cells. CAD cells were incubated with 3 µM Aβ_42_ or 0.5 µM bovine rPrP^C^ for 48 hours and washed in PBS prior to lysis. (A) Western analysis of heat shock proteins (B) The J proteins; DnaJA1(Hdj2), DnaJA2(Rdj2), DnaJA3 (Tid1) or DnaJA4 were detected by Western blot analysis. β-actin is shown as a loading control. Data are representative of 4 separate experiments.

**Figure 5** shows that in the absence of cells, heat shock *per se* does not cause degradation or oligomerization of Aβ_42._ Also, PrP^C^ does not initiate any shifts in the molecular weight of Aβ_42_ indicative of proteolysis or SDS-resistant oligomerization. In contrast a 15 kDa breakdown of PrP^C^ was observed following heat shock. **Figure 6** shows that neither PrP^C^ nor Aβ_42_ were found to alter cellular levels of the constitutive chaperones DnaJA1/Hdj2, DnaJA2/Rdj2, DnaJA3/Tid1, DnaJA4, Hsc70 or the stress induced chaperones Hsp70, Hsp40 and Hsp25, indicating that a generalized reduction in these molecular chaperone levels is not an underlying mechanism of Aβ_42_ induced neurodegeneration.

Taken together, these results indicate that heat shock chaperones have a role in clearance of cellular Aβ_42_, and may perhaps be involved in reducing Aβ_42_ pathogenesis.

### Modulation of Aβ_42_ by Hsp40 is Cell Line Specific

**Figure 7:**
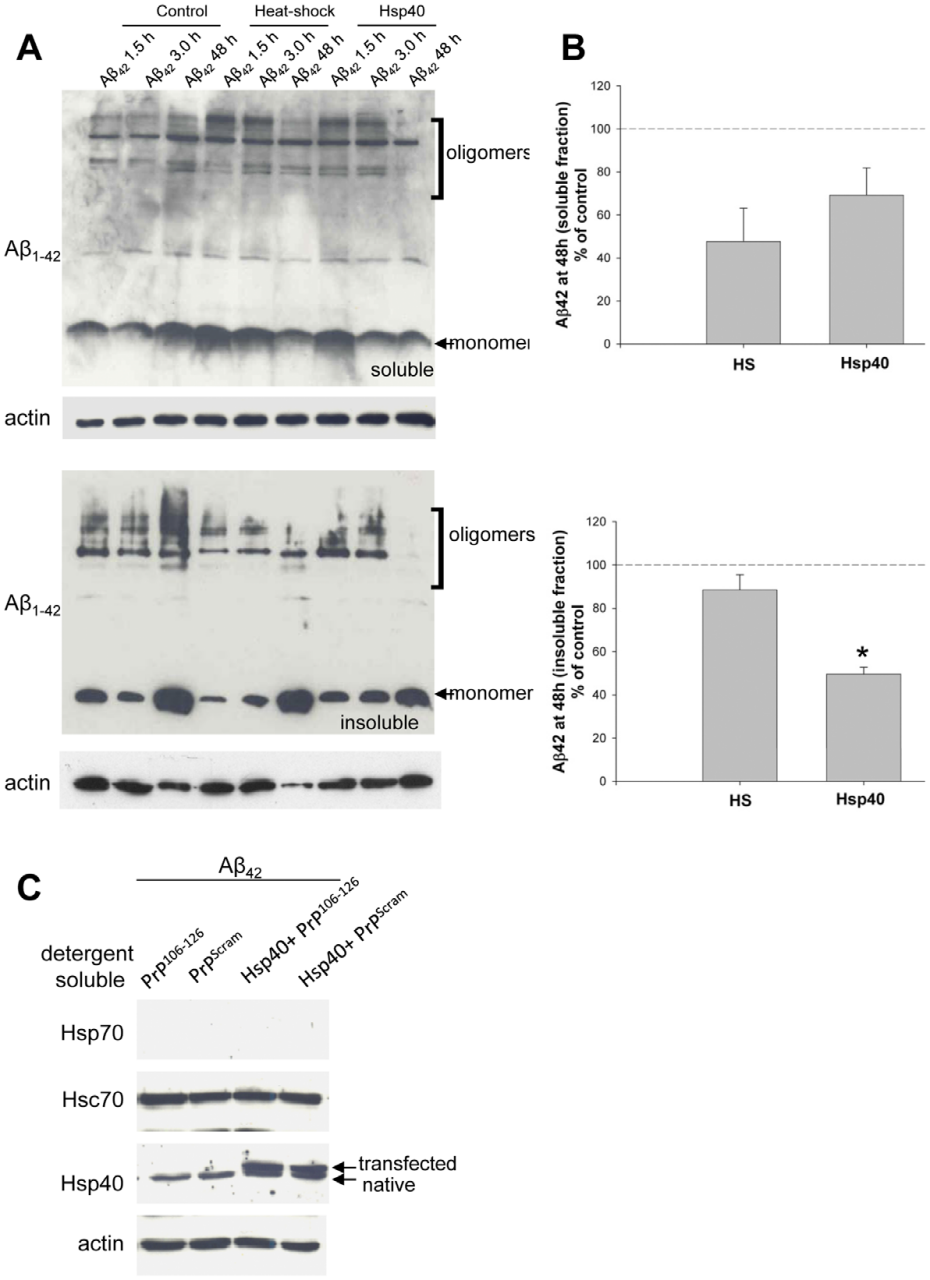
Transient transfection of Hsp40 reduces cellular Aβ_42_. (**A**) CAD cells were transiently transfected with Hsp40 as indicated for 24 hours prior to the addition of 3 µM Aβ_42_. Immediately following addition of Aβ_42_, indicated cells were subjected to heat shock at 43°C for 40 minutes and allowed to recover. At the indicated times cells were washed in PBS, lysed separated into soluble and insoluble fractions and cellular levels of Aβ_42_ were determined by Western analysis. Actin is shown as a loading control (**B**) Quantification of three independent experiments. *p<0.05. (**C**) Western analysis of CAD cells transfected with Hsp40 as indicated prior to incubation with 3 µM Aβ_42_ or 0.5 µM PrP^106−126^ or scrambled control. Data are representative of 4 separate experiments.

To further investigate the role that specific inducible chaperones play in heat shock induced reduction of Aβ_42,_ CAD cells were transfected with the stress induced J protein Hsp40 and then challenged with the toxic Aβ_42_ (**Figure 7**)_._ Both heat shock and Hsp40 transfection reduced soluble and insoluble Aβ_42_ (monomer) at 48 hours. Quantitative immunoblotting uncovered a 50% ±3 Hsp40-mediated reduction compared to a smaller heat shock-mediated decreases 88% ±6 in insoluble monomeric Aβ_42_. Soluble Aβ_42_ monomer was found to decrease to 67% ±12 following Hsp40 transfection and 48% ±15 following heat shock. Hsp40 and heat shock both caused changes in Aβ_42_ oligomerization, initially increasing the Aβ_42_ 72 kDa oligomer followed by a decrease at 48 hrs **(** **Figure 7** **)**. These experiments clearly establish Hsp40 as a chaperone that influences cellular clearance of Aβ_42_. Transfection does not induce the stress response (**Figure 7C** ). Likewise, residues encoding amino acids 106–126 of PrP^C^ as well as a scrambled control do not induce Hsp70 or increase Hsp40 levels.

**Figure 8:**
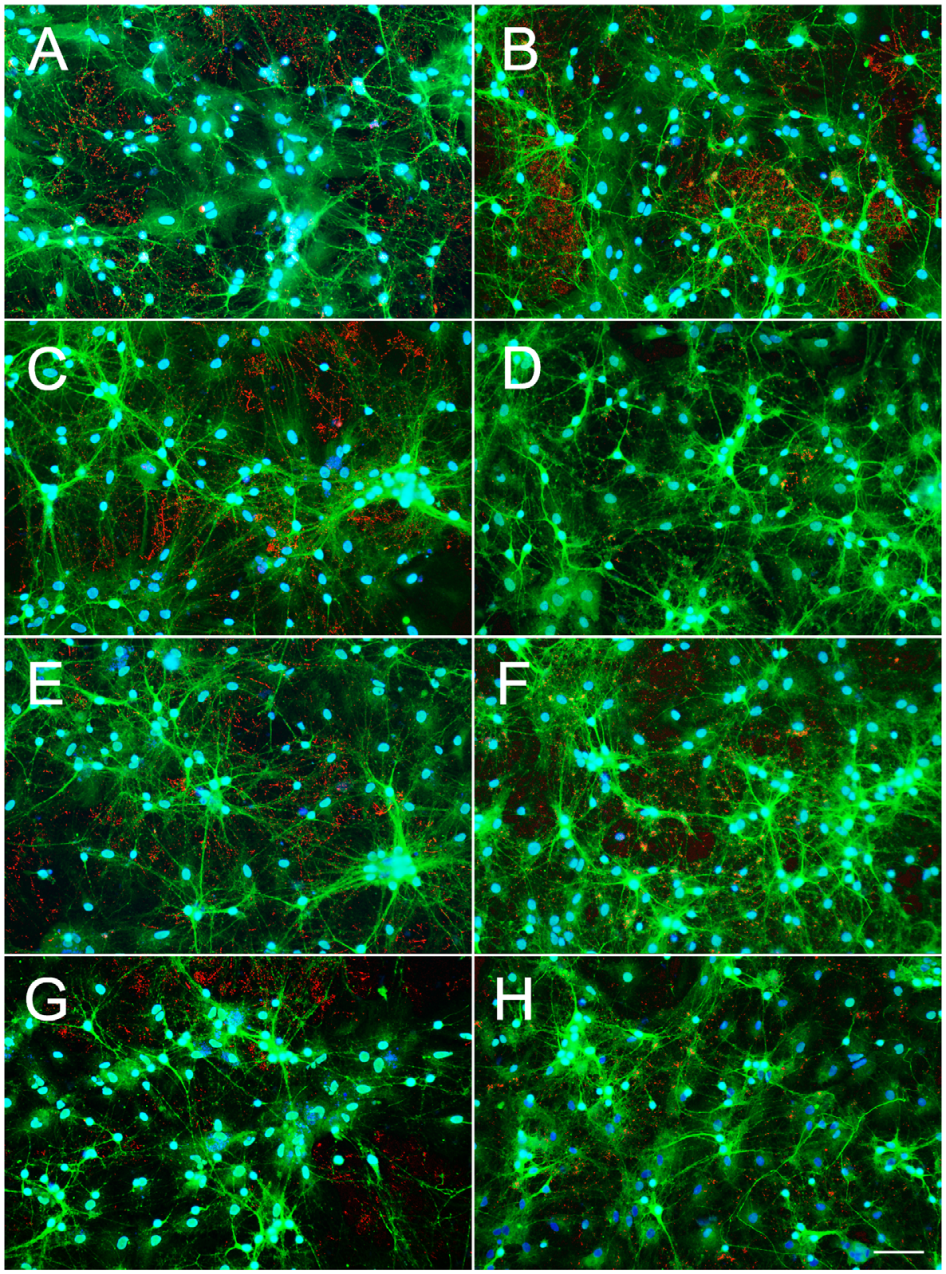
Effect of Aβ_42_ treatment on Hsp40 and Hsp40_HPD-AAA_-transfected neurons. Rat hippocampal neurons were transfected by electroporation with 4 µg myc-tagged Hsp40, Hsp40_HPD-AAA_ or vector alone and plated on silicon wafers (Silicon wafers, Silicon Quest, CA). Three days after transfection, neurons were incubated with 10 µM Aβ_42_ (panels B,D,F,H) and 7 days post transfection hippocampal cultures were fixed with 15% Picric Acid (Sigma, P6744), 4% PFA (Sigma, P6148), blocked in donkey serum/BSA and permeabilized with 0.1% Triton-X100 (Biorad, 161-0407) for 2 hours. Cultures were immunostained with Bassoon (red) (Enzo, ADI-VAM-PS003), and Neurofilament (green) (Millipore, A1991) and DAPI (blue) (Invitrogen, D1306) and examined by microscopy via an Olympus inverted scope (BX61WI) and water submergible 10x objective (UMplanF1). The panels are as follows A,B  =  controls; C,D transfected with pcDNA; E,F transfected with Hsp40 and G,H transfected with Hsp40_HPD-AAA_; A,C,E,G  =  no Aβ_42_; B,D,F,H  = 10 µM of Aβ_42_. Neurons are healthy with axon and dendritic elaboration forming networks.

**Figure 9:**
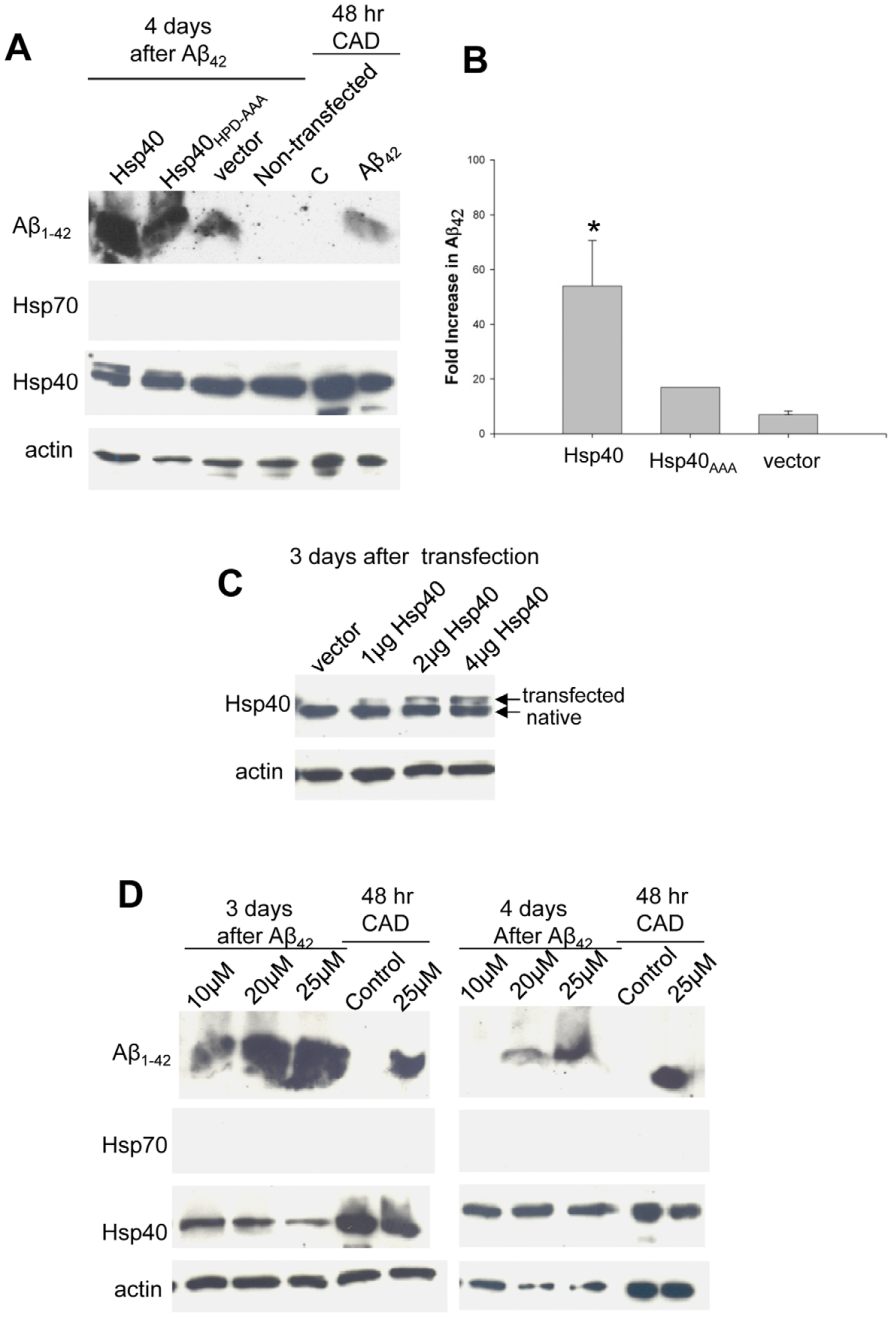
Transfection of hippocampal co-cultures with Hsp40 or Hsp40_HPD-AAA_ elevates cellular Aβ_42_. (**A**) Rat hippocampal neurons were transfected by electroporation with 4 µg myc-tagged Hsp40, Hsp40_HPD-AAA_ or vector alone. Three days after transfection, neurons were incubated with 10 µM Aβ_42_ and 7 days post transfection hippocampal cultures were washed in PBS and cellular (total) Aβ_42_ was determined by quantitative immunoblotting. Actin is shown for reference. (**B**) Quantification of three independent experiments. *p<0.01 (**C**) CAD cells were transfected with 1, 2, 4 µg myc-tagged Hsp40 as indicated. Hsp40 and actin were determined 3 days following transfection. (**D**) Western analysis of hippocampal primary cultures (3 or 4 days following external Aβ_42_ application) or CAD cells treated with Aβ_42_ for 24 hours. Data are representative of 3 separate experiments.

We then asked the question whether Hsp40 also regulates Aβ_42_ levels in primary hippocampal co-cultures of neurons and glia. Rat hippocampal neurons were isolated on postnatal day 0, transfected by electroporation with cDNA for myc-tagged Hsp40 or pCMV vector. Extensive neuritic outgrowth was found in both control and transfected cultures (**Figure 8**). Bassoon (presynaptic-red) and neurofilament (green) expressing cells are apparent. DAPI staining is shown in blue. Healthy neurons are visible in both control and Aβ_42_–treated cultures. 3 days post-transfection cultures were treated with 10 µM Aβ_42_ and 4 days later cultures were washed in PBS and cellular (total) Aβ_42_ was determined by quantitative immunoblotting. Total lysates were prepared by direct cell lysis in TX100 lysis buffer followed by SDS sample buffer to ensure that all Aβ_42_ present was evaluated by Western blot analysis. Exposure of primary hippocampal cultures to extracellular Aβ_42_ resulted in rapid Aβ_42_ clearance. **Figure 9** shows that 4 days following 10 µM Aβ_42_ exposure primary rat hippocampal cultures, which have high endogenous levels of Hsp40, reduce Aβ_42_ as does vector transfected neurons. To our surprise, in contrast to CAD neural cultures, transfection of hippocampal neurons with Hsp40 increased (59.3 fold) Aβ_42_ monomer levels over nontransfected cultures. These results reveal stark differences in neuronal processing of Aβ_42_ following Hsp40 transfection between CAD cell cultures and hippocampal cultures.

To begin to address the mechanism of the Hsp40-mediated increase in Aβ_42_ hippocampal co-cultures were transfected with a mutated form of Hsp40 predicted to bind to client proteins but not have chaperone activity. Hsp40 interacts with and activates the ATPase Hsp70/Hsc70 via its J domain. An HPD (histidine-proline-aspartic acid) motif located within the J domain of Hsp40 is required for harnessing Hsc70/Hsp70 for conformational work. By mutating the histidine-proline-aspartic acid motif of Hsp40 to alanines (Hsp40_HPD-AAA_) an Hsp40 that binds client protein but does not activate Hsc70/Hsp70 ATPase is generated. Transfection of hippocampal neurons with Hsp40_HPD-AAA_ increased Aβ_42_ monomer (15.3 fold) compared to vector control (7.9 fold) (**Figure 9A&B**). Although Hsp40_HPD-AAA_ increased cellular Aβ_42_ levels over untreated control cells (no transfection, no extracellular Aβ_42_) and vector control cultures (transfection with vector alone followed by application of extracellular Aβ_42_), the Hsp40_HPD-AAA_-mediated increase (15.3 fold) was smaller compared to Hsp40 (59.3 fold) indicating that the HPD motif within the J domain of Hsp40 impacts directly on cell processing of Aβ_42_. **Figure 9C &D** show that transfection efficiency is lower in co cultures compared to CAD cells. Like that seen for CAD cells, Aβ_42_ did not induce the heat shock response in hippocampal neurons. PrP^C^ did not block the association/accumulation of Aβ_42_ in hippocampal neurons (data not shown). **Figure 9D** compares total cellular hippocampal cultures exposed to 10, 20, 25 µM Aβ_42_ at the time of transfection (left panel) and three days following transfection (right panel) to CAD cells treated for 24 hours with 25 µM Aβ_42_.

Taken together, these results demonstrate that modulation of Aβ_42_ by Hsp40 is cell line specific.

In summary we have found that Hsp40 is able to influence cellular levels of Aβ_42._ Neural processing of extracellular Aβ_42_ is dynamic. Overproduction and impaired clearance of Aβ_42,_ are implicated in AD [8]. Accumulation of Aβ oligomers can lead to synaptic dysfunction and disruption in neural plasticity [42]–[45], however, details of the molecular cascade(s) that underlie Aβ_42_ neuronal toxicity remain unclear. Understanding how cells regulate cellular Aβ_42_ levels requires identification of the cellular chaperone machinery involved in processing the neural amyloid pool. In this study we provide mechanistic evidence that Hsp40 regulates association/accumulation of extracellular Aβ_42_ with neurons and that Hsp40-mediated regulation is cell specific.

Hsp40 is an evolutionarily ancient and widely expressed chaperone that almost certainly targets multiple client proteins. In neurons, Hsp40 is found to be concentrated in postsynaptic densities [46], in lipid rafts [47] and in association with presynaptic chaperones [38]. Furthermore, Hsp40 is linked to neurite outgrowth [48]. That said, its precise role in synaptic transmission and neurodegeneration is not yet known. Hsp40 is a member of the J protein family [49]. All J proteins have a tetrahelical Hsp70/Hsc70-interacting domain called a J domain. Via their J domain, J proteins target a wide array of cellular proteins to the ATPases Hsc70/Hsp70 for conformational work. Although our observation that Hsp40 alters Aβ_42_ cellular turnover in a cell culture specific manner is in contrast to the notion that heat shock chaperones rid the cell of toxic proteins in all cells, it is possible that in disease conditions Hsp40 may protect a toxic protein (eg Aβ_42_) from triage resulting in acceleration of disease progression. In fact, the J protein family determines which chaperone pathway is pursued by Hsc70/Hsp70 [50]. For example, DnaJB2(HSJ1) stimulates ubiquitination and sorting of substrates to the proteosome [51], while DnaJC6(auxilin) stimulates recycling of clathrin from clathrin coated vesicles [52] and DnaJB6 (Mrj) regulates keratin turnover [53]. Further experimentation is required to understand how chaperones like Hsp40 either aid in folding and maintenance or lead to degradation.

Cellular levels of Hsp40 routinely rise and fall as part of the heat shock program. Levels of constitutive Hsp40 vary drastically among neural cell lines [38] and classes of neurons in the adult rat brain [54] but how this correlates with Aβ_42_ levels remains to be established. Although the heat shock response is a highly conserved cellular program that confers transient cyto-protection via the induction and translocation of stress induced chaperones, differences in the threshold for the induction of the heat shock response, the complement of heat shock proteins induced as well as the constitutive expression of heat shock proteins are observed among neurons. For example, Hsp27 is induced by heat shock in primary hippocampal cultures but not primary cortical cultures [18]. Also, some neurons (eg motor neurons) have a high threshold for inducing the heat shock response [55]. The cellular variations in either constitutive expression or heat shock expression of Hsp40 may well lead to selective vulnerability of neurons. Other heat shock proteins have been shown to protect against Aβ_42_ neuronal toxicity. Expression of Hsp27 in cortical neuronal cultures prepared from postnatal day 1 rats protects against Aβ_42_-associated toxicity [18]. Also, Hsp70 overexpressing mice have reduced Aβ [19]. Our data show that extracellular Aβ_42_ does not elicit a heat shock response in neurons nor does it alter induction of the heat shock response. Moreover we demonstrate that while heat shock initially increases neural Aβ_42_, with time in culture Aβ_42_ levels are reduced in neurons following heat shock compared to control cultures.

The basis of the cell specific responses to Hsp40 is likely due to differences in the chaperone networks between the cultured cells. The elaborate chaperone machinery that is present in cells rids the cell of toxic proteins often via assembly of chaperones into active chaperone complexes. Levels of chaperones and chaperone complexes may differ between CAD neuroblastoma cells and primary hippocampal cultures. Further experimentation is required to develop a detailed and comprehensive overview of differences in chaperones among neurons. In conclusion we have shown that **(1)** Hsp40 modulates processing of extracellular Aβ_42._ **(2)** Hsp40 modulation of Aβ_42_ is cell specific and dependent on the conserved HPD motif within Hsp40’s J domain. **(3)** Aβ_42_ does not trigger the heat shock response or alter the threshold for induction of the heat shock response. **(4)** Soluble PrP^C^ does not block association of extracellular Aβ_42._ Overall these results elucidate an important link between Hsp40 and cellular levels of Aβ_42,_which has not been illustrated previously.

## Materials and Methods

### Reagents and Chemicals

Amyloid- β_42_ (Aβ_42_) was from rPeptide. Recombinant bovine PrP^C^ (rPrP^C^) was from Prionics. Anti-Aβ 6E10 monoclonal antibody and DnaJA4 monoclonal antibody were from Cedarlane Laboratories. Anti-Hsp40 rabbit polyclonal, anti-Hsp70 mouse monoclonal and anti-Hsp25 mouse monoclonal were from Stressgen. Anti-Hsc70 mouse monoclonal and anti-β-actin mouse monoclonal were from Sigma. Geldanamycin was from Calbiochem. Anti-Rdj2 mouse monoclonal and anti-DnaJA3 (Tid1) polyclonal antibody were from Abnova. Caspase-3 (8G10) Rabbit monoclonal antibody was from Cell Signaling Technology. DnaJ1 (Hdj2) polyclonal antibody was from USBiological. Peroxidase-conjugates AffiniPure Goat Anti-Mouse IgG (H+L) was from Cedarlane.

### CAD (CNS Catecholaminergic Derived) Mouse Neuroblastoma Cells [38], [56], [57]

CAD (CNS catecholaminergic derived) mouse neuroblastoma cells were seeded into 6 well plates and grown in DMEM/F12 medium supplemented with 10% fetal bovine serum and 1% penicillin/streptomycin as previously described. Cells were lysed in 40 mM Tris (pH 7.4), 150 mM NaCl, 2 mM EDTA, 1 mM EGTA, 1 mM Na_3_VO_4_, 0.1% SDS, 1% TX-100, 0.5 mM PMSF and protease inhibitor (Sigma) at 4°C for 1 hour. Lysates were centrifuged at 15000×g for 5 minutes at 4°C and the supernatant (soluble fraction) and pellet (insoluble fraction) was collected and stored at −70°C. For transient transfection, CAD cells were washed in PBS and transiently transfected with c-myc tagged rat Hsp40 DNA using Lipofectamine-2000 (Invitrogen) in Opti-MEM. For heat shock experiments cells were incubated at 43°C for 40 minutes and then returned to 37°C. Amyloid-β_1-42_-TFA was dissolved in 1% NH_4_OH as recommended by the supplier (rPeptide) to a concentration of 1 mg/mL. The resulting solution was sonicated for 1 minute, aliquoted and stored at −70°C. With this preparation, Aβ_42_ will be mainly in the monomeric form (∼95%) [58]. Recombinant bovine Prion Protein (Prionics, amino acids PrP^C25-242^) was suspended in H_2_0, aliquoted and stored at −70°C. PrP^C106-126^ and the scrambled PrP^C106-126^ (Anaspec) were resuspended in DMSO to a final stock concentration of 1 mM and stored at −20°C [59]. Recombinant mouse His-tagged L42 prion protein was prepared as previously described [60], [61]. Aliquots were diluted in culture media immediately prior to treatment of cells. Protein concentration of the soluble CAD cell fraction was determined by Bradford assay (BioRad). The 1% TX-100/0.1% SDS insoluble cell fraction was dissolved directly in sample buffer.

### Primary Hippocampal Cell Culture [62], [63]

Dissociated primary co-cultures of neurons and glia were isolated by dissection from Sprague Dawley rats (Charles River) at postnatal day 0 as previously described [62]. Animals were anesthetized on ice and sacrificed by decapitation. Hippocampi were removed and incubated in 40 µl/ml of papain. After 30 minutes cells were washed three times in fresh Eagle’s basal media (GIBCO-Invitrogen) supplemented with B-27, penicillin, streptomycin, L-glutamine and 4% feta bovine serum. Cells were triturated using three decreasing calibers of pipettes and the cell solution was then transfected by electroporation (Bio Rad Gene Pulsor Xcell:settings 150 volts, pulse length 25, 5 pulses, pulse interval 0.1 and cuvete size 4 mm) with cDNA of either myc-tagged Hsp40 or Hsp40_HPD-AAA_ or vector control (pCDNA3.1) and then plated. For immunostaining, transfected cells were plated on silicon wafers (Silicon wafers, Silicon Quest, CA). Co-cultures were grown for 7 days in a 5% CO_2_ incubator. After transfection, Aβ_42_ was added to the culture and 3 days later the cells were washed in PBS, lysed in 40 mM Tris (pH 7.4), 150 mM NaCl, 2 mM EDTA, 1 mM EGTA, 1 mM Na_3_VO_4_, 0.1% SDS, 1% TX-100, 0.5 mM PMSF and protease inhibitor (Sigma) at 4°C for 1 hour. Total cell lysates were solubilized directly in sample buffer and evaluated by Western blot analysis. The University of Calgary Conjoint Faculties Research Ethics Board specifically approved this study (protocol number M09008).

### Immunoblotting

Proteins were electrotransferred from polyacrylamide gels to 0.2 µm nitrocellulose membrane in 20 mM Tris, 150 mM glycine and 12% methanol. Membranes were blocked with in PBS with 0.1% Tween 20, 4% milk and incubated with primary antibody overnight at 4°C. The membranes were washed and incubated with horseradish peroxidase-coupled secondary antibody. The signal was developed using West Pico reagent (Pierce Biotechnology Inc.) and exposed to Kodak film. Bound antisera were quantitated by Biorad Fluor-S MultiImager Max and QuantityOne 4.2.1 software.
